# Supplementary material for: HIVprotI: an integrated web based platform for prediction and design of HIV proteins inhibitors
Source: J Cheminform. 2018 Mar 9;10:12. doi: 10.1186/s13321-018-0266-y (PMC5845081; doi:10.1186/s13321-018-0266-y)
Supplement: Supplementary file 2 — Additional file 2. Source code of HIVProtI web server. [file 13321_2018_266_MOESM2_ESM.zip › HIVprotI_Source-code/hivprotI/weka-3-6-6/doc/weka/classifiers/trees/j48/package-frame.html]

weka.classifiers.trees.j48


weka.classifiers.trees.j48

Classes 
  
BinC45ModelSelection
  
BinC45Split
  
C45ModelSelection
  
C45PruneableClassifierTree
  
C45PruneableClassifierTreeG
  
C45Split
  
ClassifierSplitModel
  
ClassifierTree
  
Distribution
  
EntropyBasedSplitCrit
  
EntropySplitCrit
  
GainRatioSplitCrit
  
GraftSplit
  
InfoGainSplitCrit
  
ModelSelection
  
NBTreeClassifierTree
  
NBTreeModelSelection
  
NBTreeNoSplit
  
NBTreeSplit
  
NoSplit
  
PruneableClassifierTree
  
SplitCriterion
  
Stats
